# Mediation effect of peripheral blood inflammatory factors in the association between severity of nonlactating mastitis and glycemic abnormalities

**DOI:** 10.5937/jomb0-52268

**Published:** 2025-03-21

**Authors:** Dianchun Qi, Yiqi Lin, Jiaxin Zhang, Yiping Wang

**Affiliations:** 1 Jilin Maternal and Child Hospital, Department of Breast, Changchun, China

**Keywords:** non-lactation mastitis, inflammatory cyto kines, glucose abnormality, mediating effect, mastitis bez laktacije, inflamatorni citokini, abnormalnost glukoze, posredujući efekat

## Abstract

**Background:**

The association between severity of non-lactating mastitis and glycemic abnormalities was analyzed to investigate the mediating role of peripheral inflammatory factor levels in the association.

**Methods:**

A total of 337 cases were included in this study, including 195 cases in the control group and 142 cases in the case group. Multifactorial logistic regression was used to analyze the associations between the severity of NLM and glycemic abnormality and peripheral inflammatory factors , and a mediation model was used to explore the mediating roles of the levels of hs-CRP, WBC, and IL-6 in the associations between the two.

**Results:**

The inflammatory factors IL-6, WBC, and hs-CRP had mediating effects, with effect values of 0.009 (0.003-0.038), 0.006(0.001-0.047), and 0.007(0.001-0.051), and mediating effect percentages of 2.38%, 2.12%, and 2.24%, respectively.

**Conclusions:**

NLM severity and glycemic abnormalities were positively correlated with peripheral inflammatory factors , and peripheral inflammatory factors played a partial mediating role in the association.

## Abstract (sr)

**Uvod:**

Veza između težine mastitisa bez laktacije i glikemijskih abnormalnosti je analizirana da bi se istražila posrednička uloga nivoa perifernih inflamatornih faktora u vezi.

**Metode:**

U ovu studiju je uključeno ukupno 337 slučajeva, uključujući 195 slučajeva u kontrolnoj grupi i 142 slučaja u grupi slučajeva. Multifaktorska logistička regresija je korišćena za analizu povezanosti između težine NLM-a i glikemijske abnormalnosti i perifernih inflamatornih faktora, a korišćen je model medijacije da se istraži posrednička uloga nivoa hs-CRP, VBC i IL-6 u udruženjima. između njih dvoje.

**Rezultati:**

Inflamatorni faktori IL-6, VBC i hs-CRP su imali posredne efekte, sa vrednostima efekta od 0,009 (0,003-0,038), 0,006 (0,001-0,047) i 0,007 (0,001-0,051 procenata) i posrednog efekta 2,38%, 2,12% i 2,24%, respektivno.

**Zaključak:**

Težina NLM-a i abnormalnosti glikemije bile su u pozitivnoj korelaciji sa perifernim inflamatornim faktorima, a periferni inflamatorni faktori su imali delimičnu posredničku ulogu u vezi.

## Introduction

Non-lactation mastitis (NLM) is a prevalent, benign mastitis disease in women in clinical practice [1]. It is primarily classified into two categories: periductal mastitis (PDM) and granulomatous lobular mastitis (GLM) [2] [3]. which present with a multitude of atypical clinical symptoms [4] [5]. PDM and GLM manifest similarly in clinical settings and exhibit notable histopathological similarities, necessitating histopathological diagnosis for accurate classification. The principal manifestations are vague breast pain, redness, swelling, fever, and the presence of inflammatory nodules and lumps in the breast [6]. Some patients also present with accompanied by nipple discharge, nipple inversion, and in severe cases, axillary lymph node enlargement and nodular erythema. In cases where the disease persists, the progression of the condition can result in the formation of abscesses, which may subsequently lead to the development of sinus tracts or fistulae. These complications can affect the breast in a unilateral or bilateral manner, potentially causing significant damage to the breast tissue [7]. Due to the prolonged course of the disease and its propensity for recurrence, NLM represents a significant threat to the physical and mental health of patients. It is associated with a heightened risk of developing low self-esteem, anxiety and depression [8], which has a detrimental impact on the quality of life of patients [9] [10].

NLM is mostly caused by non-bacterial inflammation, often with self-limiting and self-healing processes [11], and can occur in all periods of time, such as female infants, young adults, and the elderly, and it is more common in the age of 20–40 years old, especially within 5 years after pregnancy [12]. Although non-lactating mastitis is relatively common in women, previous studies have suggested that it may be associated with congenital malformations or dysplasia resulting in stagnation of ductal luminal secretions, bacterial infections such as Corynebacterium and Mycobacterium, hyperprolactinemia, and autoimmunity [13].

In studies of NLM, the expression of peripheral blood inflammatory factors is closely related to the severity of the disease [14]. Inflammatory factors such as tumor necrosis factor-α (TNF-α), interleukin-6 (IL-6), C-reactive protein (CRP) and other inflammatory factors may show different degrees of elevation in patients with NLM, reflecting the body’s inflammatory response to the disease. These inflammatory factors may play a mediating role in the pathophysiologic process of NLM, mediating the pathological changes of NLM by regulating the activity of immune cells and influencing the degree of inflammatory response [15] [16]. On the other hand, the relationship between dysglycemia, a common metabolic disorder, and inflammation has attracted widespread attention. It has been shown that dysglycemia may interact with inflammatory response [17]. However, the exact relationship between glycemic abnormalities and disease severity in NLM has not been clarified [18].

Therefore, this study aims to investigate the potential relationship between disease severity and glycemic abnormalities in patients with NLM and to focus on the mediating role of peripheral blood inflammatory factors in this relationship, so as to provide a new theoretical basis for the prevention, diagnosis, and treatment of NLM, and to provide a scientific guideline for the individualized treatment and precise intervention of NLM, thus improving patients’ therapeutic efficacy and quality of life; and at the same time, it is expected to identify the potential therapeutic targets [19], to provide a new direction for the future disease interventions and therapies, and to infuse the research field of the related mammary gland diseases with new scientific insights.

## Materials and methods

### Study population

A total of 142 patients with non-lactating mastitis in a tertiary general hospital from August 2022 to January 2024 were included in the study consecutively using the design scheme of prospective chart-controlled study, and a total of 195 cases from the healthy population were included in the control group. The study was approved by the ethics committee of the hospital and the subjects signed an informed consent form before all examinations.

### Inclusion and exclusion criteria

#### Inclusion criteria

(1) Age between 18 and 45 years old; (2) Meet the diagnostic criteria for non-lactating mastitis and exclude other benign and malignant tumors of the breast; (3) Patients who have not received incision and drainage, interventional therapy, radiotherapy, chemotherapy, anti-inflammatory therapy (such as hormone therapy).

#### Exclusion criteria

(1) Patients with the presence of other systemic inflammatory diseases (e.g. rheumatoid arthritis, etc.); (2) Pregnant or lactating women or patients with the presence of breastfeeding mastitis; (3) Patients with severe mental illness (such as major depression, schizophrenia, etc.).

#### Diagnostic methods

The diagnosis of non-breastfeeding mastitis usually involves physical examination, assessment of clinical symptoms and relevant laboratory tests. Non-lactating mastitis is diagnosed when clinical symptoms include breast pain, swelling, and fever, and when the location and size of the lesion can be recognized by breast ultrasound or mammography or breast MRI. Inflammatory indicators were measured in the laboratory by serologic Enzyme-linked Immuno Sorbent Assay (ELISA) method, and specific inflammatory factors including white blood cell (WBC), IL-6, and hs-CRP were mainly selected for the study. WBC, IL-6 and hs-CRP function both as inflammatory factors and inflammatory markers. Inflammatory factors are involved in the inflammatory process (as factors) and may also be measured to assess inflammation (as markers).

Regarding the severity of NLM, it was classified as mild mastitis (manifested by mild localized swelling, pain, and redness of the breast), moderate mastitis (manifested by increased breast pain and swelling, and a noticeable hard lump of breast tissue), and severe mastitis (accompanied by breast abscesses, sinus tracts, or fistulae formation).

#### Covariates

In this study, data related to socio-demographic characteristics, history of previous illnesses, laboratory tests, and environmental exposures were collected using a general information questionnaire, the hospital electronic medical record system, and laboratory test data. The demographic characteristics included: age, body mass index (BMI), smoking and passive smoking status, history of diabetes mellitus, history of hypertension, drinking behavior, and duration of breastfeeding.

### Statistical analysis

International Business Machines Corporation (IBM) Statistic Package for Social Science (SPSS) 23.0 software (IBM, Armonk, NY, USA) was used for statistical analysis. Continuous variables were expressed as mean ± standard deviation and categorical variables were expressed as counts and percentages. Comparisons between two groups of continuous variables were made by t-test, and differences between two groups of categorical variables were made by χ^2^ test or Fisher’s exact test. Regression analysis method and mediation role analysis were used to assess the association and mediation role between inflammatory factors such as IL-6 and disease severity in non-lactating mastitis patients. The mediation analysis method was based on the Process 3.3 plug-in of SPSS 23.0 software to construct the corresponding regression model, and the mediation analysis was carried out using the Bootstrap test program, and the mediation effect was converted to the corresponding odds ration (OR) size using the inverse logarithmic transformation. All statistical tests were two-sided tests, with P<0.05 suggesting that the difference was statistically significant, and the test level α=0.05.

## Results

### Baseline characteristics

A total of 337 cases were included in this study, including 195 cases in the control group and 142 cases in the case group (Table 1). The age of the control group was lower than that of the NLM group, and the differences between the two groups in terms of age, history of diabetes mellitus, history of dysglycemia, history of smoking, fasting blood glucose, WBC, IL-6, and hs-CRP were statistically significant (P<0.05).

**Table 1:** Comparison of baseline information.

	Control group (n=195)	NLM group (n=142)	Statistics value	P-value
Age	26.71±3.62	27.13±3.25	t=1.057	P=0.021
BMI	21.45±3.11	21.83±3.07	t=3.082	P=0.572
History of diabetes	52	78	X^2^=3.88	P<0.001
History of hypertension	43	55	X^2^=1.29	P=0.281
History of dysglycemia	49	82	X^2^=7.284	P=0.004
History of smoking	67	62	X^2^=5.208	P=0.045
History of alcohol consumption	41	37	X^2^=3.283	P=0.501
Breastfeeding duration			X^2^=4.162	P=0.305
<1 month	85	69		
1-4 months	38	26		
>4 months	72	47		
Fasting blood glucose	5.88±1.64	6.47±2.43	t=4.506	P<0.001
hs-CRP	149.82±82.36	157.35±90.21	t=2.981	P<0.001
WBC	6.15±1.67	6.58±2.09	t=1.773	P=0.017
IL-6	149.84±80.23	156.42±89.46	t=2.002	P=0.031

### Logistic regression of the association between inflammatory factors, glycemic abnormalities and NLM severity

In order to consider the effects of confounding factors as much as possible, all factors with P<0.05 in the one-way logistic regression analysis were included in the multifactorial logistic regression analysis (Table 2). The results of the multifactorial logistic regression analysis of the association between blood glucose abnormality, inflammatory factors and disease severity showed that there was a positive correlation between blood glucose abnormality (β=0.470, P<0.001), inflammatory factors (β=0.183, P=0.010), and disease severity. The value of b represents the standardised regression coefficient, which is a measure of the magnitude of the effect of the independent variable on the dependent variable. A larger value of β indicates that the corresponding independent variable has a more significant effect on the dependent variable. This analysis considers not only the direct effect of the independent variables on the dependent variable, but also the interaction between the independent variables, thus providing a more comprehensive analysis. The p-value of all these results is less than 0.05 and the results are statistically significant. The P-value of all these results is less than 0.05 and the results are statistically significant. Simple linear regression and multifactorial linear regression were used to analyze the association between blood glucose abnormalities and peripheral inflammatory factors. Simple linear regression analysis revealed that age was correlated with the level of inflammatory factors, and after adjusting for the age factor in multifactorial linear regression analysis, the results showed that there was also an association between blood glucose abnormalities and the level of peripheral inflammatory factors (β=0.146, P= 0.023).

**Table 2:** Logistic regression of the association between inflammatory factors, glycemic abnormalities and NLM severity.

Variable	Glucose Abnormalities Linked to<br>NLM Severity		Inflammatory Factors Associated<br>with NLM Severity		Glycemic Abnormalities and<br>Inflammatory Factors	
	β	P	β	P	β	P
Age	0.133	0.087	0.135	0.081	0.074	0.083
History of diabetes	0.374	0.046	0.372	0.049	0.050	0.011
History of dysglycemia	0.470	P<0.001	0.439	P<0.001	0.146	0.023
History of smoking	0.264	0.071	0.274	0.052	0.020	0.751
Fasting glucose	0.128	0.058	0.107	0.073	0.194	0.047
Inflammatory factors	0.156	0.032	0.183	0. 010	0.038	0.016

### Analysis of mediating roles

**Figure 1:**
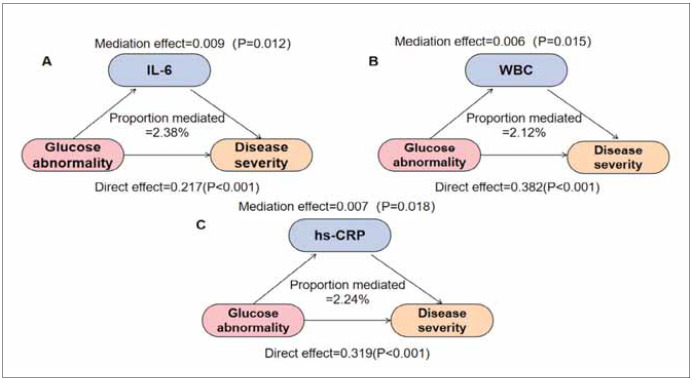
Path diagram of mediation analysis. (Note: Figure 1 represents the mediation analysis path diagram of inflammatory factors on the relationship between glycemic abnormalities and disease severity. A-C represent the mediating effects of IL-6, WBC and hs-CRP, respectively).

In terms of inflammatory effects, we explored the mediating role of each inflammatory factor (Figure 1). The results of mediation analysis showed that IL-6, WBC and hs-CRP had mediating effects, with effect values of 0.009 (0.003–0.038), 0.006 (0.001–0.047), and 0.007 (0.001–0.051), respectively (Table 3). And significantly mediated the correlation between glycemic abnormalities and disease severity, with IL-6, WBC, and hs-CRP explaining 2.38%, 2.12%, and 2.24% of the correlation, respectively (P<0.05). Although the direct effects of all three inflammatory factors were significant (P<0.001), the mediating effects of inflammatory markers tended to be nonsignificant. IL-6, WBC and hs-CRP shows a small mediation effect, with an OR close to 1, suggesting limited mediation. We found that higher inflammatory factors were significantly associated with disease severity in the included population (Table 3). The mediating effects of IL-6 (OR=1.14, 95% CI: 1.05–1.27), WBC (OR=1.02, 95% CI: 1.00–1.05), and hs-CRP (OR=1.08, 95% CI: 0.99–1.08), while statistically significant, are modest in size. These findings suggest that while these inflammatory factors contribute to the relationship between glycemic abnormality and disease severity in non-lactating mastitis, other mediating pathways likely exist. Further research is needed to fully elucidate the complex interplay between metabolic and inflammatory processes in this condition.

**Table 3:** Analysis of the mediating role of inflammatory factors in the association between dysglycemia and disease severity.

Type of<br>effect	hs-CRP			WBC			IL-6		
	Efficacy value<br>(95% CI)	Boot<br>SE	OR<br>(95% CI)	Efficacy value<br>(95% CI)	Boot<br>SE	OR<br>(95% CI)	Efficacy value<br>(95% CI)	Boot<br>SE	OR<br>(95% CI)
Total effect	0.328<br>(0.165–0.662)	0.130	1.50<br>(1.09–2.00)	0.431<br>(0.199–0.668)	0.125	1.53<br>(1.23–2.14)	0.385<br>(0.182–0.690)	0.138	1.59<br>(1.22–2.07)
Direct<br>effect	0.217<br>(0.126–0.493)	0.128	1.43<br>(1.20–1.94)	0.382<br>(0.165–0.682)	0.119	1.49<br>(1.37–1.89)	0.319<br>(0.150–0.526)	0.124	1.52<br>(1.15–1.95)
Mediation<br>effect	0.007<br>(0.001–0.051)	0.013	1.08<br>(0.99–1.08)	0.006<br>(0.001–0.047)	0.015	1.02<br>(1.00–1.05)	0.009<br>(0.003–0.038)	0.021	1.14<br>(1.05–1.27)

The total effect shows that dysglycemia is significantly associated with increased disease severity. This association is only partially mediated by inflammatory factors, as seen in the mediation effect. The direct effect reveals that even after accounting for inflammation, dysglycemia still has a significant impact on disease severity, which means that other factors aside from inflammation also contribute to this relationship. Mediation Effects are statistically significant, indicating that while inflammation plays a role in the relationship between dysglycemia and disease severity, it is not the sole pathway.

## Discussion

NLM is a common breast disease, and in recent years, the incidence rate has shown a significant upward trend, and its poor therapeutic efficacy and high recurrence rate have attracted extensive attention in the field of public health [20] [21]. Studies have shown that the onset and progression of NLM are related to the abnormal expression of inflammatory factors, especially some of them can mediate the targeted migration of target cells to the effector sites, thus affecting the immune function of the body [22]. In addition, genetics, lifestyle, infections, smoking and underlying diseases can exacerbate the severity of NLM [23]. In this pathway, fluctuations in blood glucose levels may play a key role in the pathologic process of NLM [24] [25]. A hyperglycemic state exacerbates the inflammatory response, which in turn affects the immune function of breast tissue, leading to the progression of NLM to a more severe state [26] [27]. Therefore, in addition to focusing on the role of inflammatory factors, the glycemic status of patients needs to be fully considered for a more comprehensive understanding of the pathomechanisms of NLM [28].

The multifactorial logistic regression analysis in this study revealed the complex and close interplaybetween inflammatory factors, glycemic abnormalities, and disease severity in the mechanism of NLM development. The findings not only indicated a positive association between inflammatory factors and glycemic abnormalities, but also a tendency for this association to be positively regulated with NLM disease severity. Abnormal expression of inflammatory factors may trigger the pathogenesis of NLM, leading to changes in hormone levels (e.g., prolactin, epinephrine, etc.) [29], which in turn may have an impact on blood glucose regulation [30]. Similarly, blood glucose abnormalities may further exacerbate disease severity in NLM by exacerbating the inflammatory response, interfering with immune function, and other pathways. This interaction emphasizes the existence of a bidirectional regulatory relationship between inflammatory factors and blood glucose abnormalities in the pathology of NLM, which together regulate the development of the disease [31].

In addition to this, the present study found that IL-6, WBC and hs-CRP played significant mediating roles between glycemic abnormality and the severity of non-lactating mastitis. IL-6, as an immunomodulatory factor, may mediate the effect of glycemic abnormality on mastitis by activating the inflammatory response. WBC reflects the degree of activation of the immune system of the organism, and its mediating role may reflect the WBC reflects the degree of activation of the body’s immune system, and its mediating role may reflect the sensitivity of the immune system to glycemic abnormalities. hs-CRP, on the other hand, is an inflammatory marker, and its mediating role may reflect the regulation of the inflammatory state during the development of mastitis by glycemic abnormalities.

IL-6, WBC and hs-CRP explained 2.38%, 2.12% and 2.24% of the correlation, respectively, a result that suggests that the contribution of these inflammatory factors in mediating the process is relatively small. This may indicate that the mediating role of inflammatory factors in the development of non-lactating mastitis is a complex and multifactorial process. Although the mediating effects of these inflammatory factors were relatively small, their significance still supports their independent role in modulating the relationship between glycemic abnormalities and disease severity.

In summary, there is a potential association between NLM severity and glycemic abnormalities,and peripheral inflammatory factors have a mediating role in this association. Future studies should delve into the detailed mechanism of action of peripheral inflammatory factors in order to provide more precise and effective intervention and treatment protocols, and to provide a scientific basis for early prevention and individualized treatment of NLM patients. However, the study still suffers from the following shortcomings: (1) Although confounding variables that have an impact on the study were included in this study as much as possible, there are still potential residual confounding factors that were not included in the analysis. (2) The included samples were all from a single hospital, and some of the cases were current cases, so there was some selective bias. (3) The number of cases included in this study was small, and further expansion of the sample size is needed for future studies.

## Dodatak

### Acknowledgements

Not applicable.

### Author contributions

Dianchun Qi and Yiping Wang designed the study and performed the experiments, Yiqi Lin collected the data, Jiaxin Zhang analyzed the data, Dianchun Qi and Yiping Wang prepared the manuscript. All authors read and approved the final manuscript.

### Data availability statement

The data that support the findings of this study are available from the corresponding author upon reasonable request.

### Funding

This study did not receive any funding in any form.

### Conflict of interest statement

All the authors declare that they have no conflict of interest in this work.
